# fMRI Evidence of Magnitude Manipulation during Numerical Order Processing in Congenitally Deaf Signers

**DOI:** 10.1155/2018/2576047

**Published:** 2018-12-18

**Authors:** Josefine Andin, Peter Fransson, Jerker Rönnberg, Mary Rudner

**Affiliations:** ^1^Linnaeus Centre HEAD, Swedish Institute for Disability Research, Department of Behavioural Sciences and Learning, Linköping University, SE-581 83 Linköping, Sweden; ^2^Department of Clinical Neuroscience, Karolinska Institute, SE-171 77 Stockholm, Sweden

## Abstract

Congenital deafness is often compensated by early sign language use leading to typical language development with corresponding neural underpinnings. However, deaf individuals are frequently reported to have poorer numerical abilities than hearing individuals and it is not known whether the underlying neuronal networks differ between groups. In the present study, adult deaf signers and hearing nonsigners performed a digit and letter order tasks, during functional magnetic resonance imaging. We found the neuronal networks recruited in the two tasks to be generally similar across groups, with significant activation in the dorsal visual stream for the letter order task, suggesting letter identification and position encoding. For the digit order task, no significant activation was found for either of the two groups. Region of interest analyses on parietal numerical processing regions revealed different patterns of activation across groups. Importantly, deaf signers showed significant activation in the right horizontal portion of the intraparietal sulcus for the digit order task, suggesting engagement of magnitude manipulation during numerical order processing in this group.

## 1. Introduction

Numerical processing abilities are closely associated with mathematical success [1], and ordinal relationships have been suggested to be important for efficient number processing [2]. In general, the literature suggests that deaf individuals often have poorer mathematical abilities than their hearing peers [3, 4]. However, in a recent study, we have shown that when groups are carefully matched on age, education, and nonverbal intelligence, arithmetic abilities are similar between groups [5]. It is not known whether the same neural networks underpin numerical order processing in deaf signers and hearing nonsigners. In the present study, we investigated this. For deaf children born into deaf families, sign language is the natural form of communication [6]. Signed languages are complete natural languages that have their own vocabulary and grammar, and developmental milestones are reached in the same order for signed and spoken languages [6–8]. Deaf people also use manual systems for representing numerals and letters of the alphabet [9]. Despite the often reported discrepancy in mathematical skills between deaf and hearing individuals, there is less evidence of differences in the fundamental numerical skills of subitizing [10], magnitude processing [10], and number comparisons [3]. Indeed, deaf children have been shown to perform better than hearing children on spatial tasks [11] and nonsymbolic subtraction tasks [12]. Their problems seem to be more specifically related to numerical processing that requires more abstract manipulation involving linguistic representations. This has been shown to apply in particular to relational statements (e.g., less than, more than, and twice as many as) [13, 14], arithmetic problems that require reading [15], fractions [16], and multiplication [5, 17]. It has been suggested that the establishment of verbal number representations in deaf individuals might be altered or delayed, due to weaker associations between concepts and a high reliance on item-specific, compared to relational processing [18, 19]. Only one previous imaging study to our knowledge has investigated the neural correlates of numerical processing in deaf signers [20]. Results showed that learning the numerals of a new sign language activates the numerical processing networks established for hearing individuals. However, we are not aware of any previous studies investigating numerosity judgment or numerical order processing in deaf signers. Findings from brain imaging suggest that numerosity judgment in hearing individuals engages bilateral parietal regions. Activation of the posterior superior parietal cortex occurs during number comparison [21], approximation [22], and counting [23]. However, this region has been shown to be activated during a range of visuospatial tasks including mental rotation, spatial working memory, and attention orienting [24, 25]. Thus, it is not reserved for numerical processing. Numerical processing also activates the left angular gyrus when verbal coding and processing are required. Thus, a greater activation of this region has been found for exact, compared to approximate, calculation [22], for small, compared to larger, digits [26], for multiplication compared to addition [27] and subtraction [28], and for addition compared to subtraction [29]. The bilateral horizontal portion of the intraparietal sulcus is thought to be the locus of magnitude manipulation and thus the mental number line [30, 31]. Activation in this region has been reported for subtraction compared to multiplication [32, 33], for approximate compared to exact calculation [22], and for number words compared to other words [34]. It has also been suggested that the function of this region is specific to magnitude manipulation [33]. In the present study, we investigated numerical order processing in deaf signers and hearing nonsigners, well-matched on age, education, and nonverbal intelligence, by presenting a digit order task during functional magnetic resonance imaging (fMRI). In order to determine whether activation patterns were specific to digit ordering, rather than ordering in general, we also administered a letter order task based on the same stimulus material, which consisted of sets of three printed digit/letter pairs. Activation relating simply to visual stimulation engendered by the stimuli was subtracted using a visual control task also based on the same stimulus material. Four hypotheses were tested: (1) At a whole brain level, there will be general similarities between groups for both the digit and the letter order task. (2) For the digit order task, there will be bilateral activation in regions of interest including the superior parietal lobule (SPL) and the horizontal portion of the intraparietal sulcus (hIPS), as well as in the left angular gyrus (lAG) for both groups. (3) Activation for the digit order task will be greater than that for the letter order task in hIPS, reflecting the magnitude manipulation specificity of this region, generalizing it to deaf signers. (4) Hearing nonsigners will show greater activation in the lAG compared to deaf signers for both digit order and letter order, reflecting differential engagement of linguistic representations.

## 2. Methods

### 2.1. Participants

The study included 16 deaf adults (*M* = 28.1 years, SD = 3.44, range 21–32; eleven women) and 17 native Swedish-speaking hearing adults (*M* = 28.6 years, SD = 4.85, range 22–37, twelve women, one of whom was excluded; see Data Analysis, leaving 16 hearing nonsigners). The participants were all right handed and reported at least 12 years of formal schooling (equivalent to high school degree). Five participants in each group had a university degree or equivalent level of education. Intelligence was screened using Raven's standard progressive matrices. This resulted in no statistically significant differences between groups in age (*t*(30) = 0.166, *p* = 0.869), nonverbal IQ (*t*(30) = 1.512, *p* = 0.141), or educational level (high school vs. university degree; *χ*^2^(32) = 0.00, *p* = 1.00; Table 1). Participants had normal, or corrected-to-normal, vision and reported no neurological or psychiatric illnesses. They also complied with the following exclusion criteria: pregnancy, claustrophobia, medications (except for contraceptives), and non-MRI compatible metal implants.

Fifteen of the deaf participants were deaf from birth and one from the age of six months. All reported using Swedish Sign Language (SSL) daily as their primary language. Six were exposed to SSL from birth and the others before the age of two.

The prevalence of congenital deafness is around 1 in a thousand live births, and only 5% of congenitally deaf children are born into signing families. Thus, deaf early signers constitute a very small population. Further, many deaf signers are opting for cochlear implantation which is a counter indication for fMRI. The group of deaf participants in the present study is similar in size to, or larger than, those in many other studies (cf. 11 deaf participants in Emmorey et al.'s study [35]; 7 deaf participants in MacSweeney et al.'s study [36]). It is also unusually homogenous in terms of education, a factor that is often not reported. It would have been preferable to have a larger group, but this was not possible due to demographic constraints.

All participants gave written informed consent and were compensated for time and travel expenses. Approval was obtained from the regional ethical review board in Linköping, Sweden (Dnr 190/05).

### 2.2. Stimuli and Tasks

Stimuli were identical across tasks and the control condition. They consisted of sets of three-digit/letter pairs, e.g., V2 X5 U7. The pairs included the digits 0–9 and the letters B, D, E, G, H, K, L, M, O, P, Q, T, U, V, X, and Z, as well as the characters Å and Ö that are listed at the end of the Swedish alphabet. There were 20 unique sets of pairs. Each pair was also reversed within each set, e.g., 2V 5X 7U, giving 40 unique stimuli. It is important to note that the digit/letter order within pairs was never mixed within stimuli. Further, congruent (the same correct response for both tasks) and noncongruent (different correct responses for the two tasks) trials were balanced. Participants completed six different tasks, of which three are investigated in the present study. Those three tasks were digit order (“are the presented digits in an ascending numerical order?”), letter order (“are the presented letters in an alphabetical order?”), and visual control task (“are there two dots over any of the presented letters?”). Correct responses were 50% “yes” and 50% “no,” distributed orthogonally across conditions. Results from the three remaining tasks (multiplication, subtraction, and phonological similarity) are reported in two articles (Andin et al. [37]; under revision) with the digit order task serving as a baseline for the arithmetic tasks and the letter order task as a baseline for the phonological task. In neither of these articles are the ordering tasks considered in their own right.

### 2.3. Procedure

All participants took part in a behavioural testing session at least one month prior to the fMRI session for task familiarization and to ensure compliance during scanning. Before entering the scanner, participants practiced the tasks again and were instructed to respond as accurately and as quickly as possible during the presentation of each trial, by pressing one of two buttons using their right thumb and index finger. A professional accredited sign language interpreter provided deaf participants with a verbatim translation of test instructions and remained on hand to relay questions and answers. When participants were installed in the scanner, instructions were repeated again, orally for hearing individuals and as text on the screen for the deaf participants.

In the scanner, participants viewed the screen through an angled mirror on top of the head coil. Stimuli were presented using the Presentation software (Presentation version 10.2, Neurobehavioral Systems Inc., Albany, CA) and back projected onto a screen positioned at the feet of the participant. Each trial started with a 1000 ms period during which a cue displayed on the screen indicated which task was to be performed next. The cues were “1 2 3” for digit order, “a b c” for letter order, and “..” for the control task. After the cue, the stimulus was displayed for 4000 ms while the participant responded. Task presentation was blocked, and there were five trials per block. Thus, each block lasted for 25,000 ms. Between blocks, there was a 5000 ms break and a ¤ symbol was presented. Participants were instructed to move as little as possible. In total, there were 4 runs with 12 blocks in each. Of the 12 blocks, six blocks (two per condition) were considered in the present analysis.

### 2.4. Data Acquisition

Functional gradient-echo EPI images (repetition time (TR) = 2500 ms, echo time (TE) = 40 ms, field of view (FOV) = 220 × 220 mm, flip angle = 90 deg, in-plane resolution of 3.5 × 3.5 mm, slice thickness of 4.5 mm, slice gap of 0.5 mm, with enough axial slices to cover the whole brain) were acquired on a 1.5 T GE Instruments scanner (General Electric Company, Fairfield, CT, USA) equipped with a standard eight-element head coil, at the Karolinska Institute. The initial ten-second fixation period without task presentation was discarded to allow for T1-equilibrium processes. Anatomical images were collected using a fast spoiled gradient echo sequence, at the end of the scanning session (voxel size 0.8 × 0.8 × 1.5 mm, TR = 24 ms, TE = 6 ms).

### 2.5. Data Analysis

Data quality was checked using TSDiffAna (Freiburg Brain Imaging). As a result, the first run was discarded for three deaf participants and one hearing participant who moved more than 3 mm in at least one direction. Remaining data was preprocessed and analysed using statistical parametric mapping software (SPM8; Wellcome Trust Centre for Neuroimaging, London, UK) running under MatLab r2010a (MathWorks Inc., Natick, MA, USA). Preprocessing included realignment, coregistration, normalization to the MNI152 template, and spatial smoothing using a 10 mm FWHM Gaussian kernel, following standard SPM8 procedures.

Blocks with more than two incorrect answers were discarded from the analysis (two-letter order blocks and one-digit order block were removed from the hearing group, and two-letter order blocks were removed from the deaf group), because the response pattern in some cases suggested nonadherence to the task. Data from one hearing participant were removed due to artefacts probably caused by metallic hair dye. Thus, data from 16 participants in each group were included in the functional analysis. Analysis was conducted by fitting a general linear model (GLM) with regressors representing each of the two experimental conditions of interest here (digit order and letter order) and the visual control, as well as the six motion parameters derived from the realignment procedure. At the first-level analysis, contrast images consisting of digit order versus visual control and letter order versus visual control were defined individually for each participant. To investigate hypothesis 1 that there will be general similarities between groups for both the digit and the letter order task at the whole brain level, the contrast images from the first level analysis were brought into second-level analyses where sample *t*-tests for the two groups separately and one for the two groups together were performed. The presence of any group differences was tested for using two independent sample *t*-tests, one for letter order and one for digit order. The significance was determined using family-wise error (FWE) correction for multiple comparisons at *p* < 0.05 for the voxel level for peak values for the whole brain. Images were prepared using SPM8 and MRIcron software (ver. 6/2013, McCausland Center for Brain Imaging, Columbia, USA).

Hypotheses 2–4 were investigated using separate region of interest analyses (ROI) for the left angular gyrus (lAG), left and right superior parietal lobules (lSPL and rSPL), and left and right horizontal portions of the intraparietal sulcus (lHIPS and rHIPS; using the toolbox MarsBar, release 0.44). The ROIs were defined in accordance with the probabilistic cytoarchitectonic maps from an SPM anatomy toolbox (version 1.8). To investigate hypothesis 2 that for the digit order task there will be activation in all five ROIs for both groups, group level contrasts were obtained to determine significant activation within the five ROIs. Further, to investigate the final two hypotheses that activation for the digit order task will be greater than that for the letter order task in hIPS (hypothesis 3) and that hearing nonsigners will show greater activation in the lAG compared to deaf signers for both digit order and letter order (hypothesis 4), individual contrast values from the HIPS and lAG of the ROI analysis were extracted for further statistical analyses. These analyses were carried out as a 2 × 2 × 2(task [digit order, letter order] × hemisphere [right, left] × group [deaf signers, hearing nonsigners]) analysis of variance in HIPS (hypothesis 3) and as an independent *t*-test in the lAG (hypothesis 4). The analyses of contrast values from the ROI analyses as well as the in-scanner response time and accuracy data analyses were performed using SPSS statistics 22 (IBM, SPSS Statistics, version 22, IBM Corporation, New York, USA). The design of the analyses of variance for response time and accuracy was 2 × 2(task [digit order, letter order] × group [deaf signers, hearing nonsigners]). As for the imaging data, blocks with more than two incorrect answers were removed from the behavioural analysis.

## 3. Results

### 3.1. Behavioural Data

Behavioural data are shown in Table 2. Response time data revealed the main effect of task (*F*(1, 30) = 308.4, *p* < 0.001, partial *ɳ*^2^ = 0.911). This showed that the digit order task (*M* = 1608 ms, SD = 1847) was performed faster than the letter order task (*M* = 2344 ms, SD = 234). There was no main effect of the group (*F*(1, 30) = 0.004, *p* = 0.952, partial *ɳ*^2^ = 0.000) (deaf signers: *M* = 1979 ms, SD = 240; hearing nonsigners: *M* = 1973 ms, SD = 304), and there was no group × task interaction (*F*(1, 30) = 0.006, *p* = 0.937, partial *ɳ*^2^ = 0).

As with the response time data, accuracy data revealed a main effect of task (*F*(1, 30) = 22.4, *p* < 0.001, partial *ɳ*^2^ = 0.428). This showed that performance on digit order was more accurate (*M* = 97.2%, SD = 3.56) than that on letter order (*M* = 91.4%, SD = 3.14). Again, there was no main effect of the group (*F*(1, 30) = 0.988, *p* = 0.328, partial *ɳ*^2^ = 0.032) (deaf signers: *M* = 93.8%, SD = 4.13; hearing nonsigners: *M* = 94.9%, SD = 1.78) and no group × task interaction (*F*(1, 30) = 1.49, *p* = 0.231, partial *ɳ*^2^ = 0.047).

### 3.2. Imaging Data

#### 3.2.1. Whole Brain Analyses

The results of the whole brain analysis to test hypothesis 1 are shown in Table 3. Neither group showed any significant activation for the digit order contrast. For the letter order contrast, the deaf group showed significant activation in the right occipital gyrus (however, only at the cluster level, not at the peak level) and the hearing group showed significant activation in the frontal, parietal, and occipital regions. Although the activation for the hearing group was more widespread than that for the deaf group, independent *t*-tests showed no significant group effects for either contrast.

**Figure 1:**
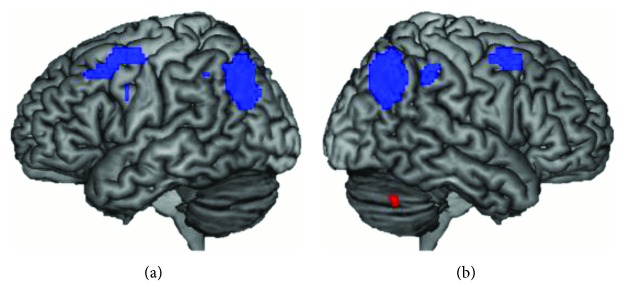
Activation pattern for digit order (red) and letter order (blue) in (a) left and (b) right hemispheres for both groups combined. Images are thresholded at a FWE-corrected *p* values of <0.05.

Because the groups show similar activation for both tasks, combining them might give additional information otherwise obscured by the low number of subjects. Therefore, the activation patterns for the two tasks were further investigated by collapsing across groups (cf. Mayer et al. [38]). The general activation pattern for both groups combined revealed a peak activation for digit order in the right cerebellum and activation in the bilateral frontal, parietal, and occipital regions for letter order (Figure 1). These regions included left lateralized activation in the superior parietal lobule, supplementary motor area, and precentral gyrus; right lateralized activation in the superior occipital gyrus; and activation in the bilateral middle occipital gyrus, superior frontal gyrus, and inferior parietal sulcus (Table 3).

#### 3.2.2. Region of Interest Analyses

To investigate our region-specific hypotheses, we analysed the variations in brain activity associated with the digit and letter order tasks in the two groups within the bilateral superior parietal lobule (SPL) and horizontal portion of the intraparietal sulcus (HIPS) as well as the left angular gyrus (lAG). In line with the second hypothesis, the digit order task significantly activated the rHIPS for deaf signers (*t* = 1.76, *p* = 0.05). However, this task did not significantly activate this region for the hearing nonsigners or any of the other regions of interest for either group. Letter order, on the other hand, showed significant activation for both groups in the rSPL (deaf signers: *t* = 2.08, *p* = 0.028; hearing nonsigners: *t* = 4.97, *p* < 0.001), lHIPS (deaf signers: *t* = 2.56, *p* = 0.011; hearing nonsigners: *t* = 5.15, *p* < 0.001), and rHIPS (deaf signers: *t* = 3.50, *p* = 0.001; hearing nonsigners: *t* = 5.34, *p* < 0.001) as well as in the lSPL for hearing nonsigners (*t* = 4.57, *p* < 0.001).

Regarding our third hypothesis, we performed a mixed design 2 × 2 × 2 ANOVA and found a significant main effect of task (*F*(1, 30) = 8.68, *p* = 0.006, partial *ɳ*^2^ = 0.224) in HIPS. However, contrary to our prediction, letter order generated greater activation than digit order in both hemispheres (left *F*(1, 30) = 7.41, *p* = 0.011, partial *ɳ*^2^ = 0.198; right *F*(1, 30) = 10.9, *p* = 0.003, partial *ɳ*^2^ = 0.266). There was also a significant main effect in the hemisphere (*F*(1, 30) = 9.30, *p* = 0.005, partial *ɳ*^2^ = 0.237) with greater activation in the right hemisphere. There was no significant main effect of the group (*F*(1, 30) = 0.79, *p* = 0.79, partial *ɳ*^2^ = 0.002).

Finally, we did not find support for our fourth hypothesis of a significant difference between groups on the digit order task within the lAG (*t*(30) = 0.159, *p* = 0.875).

## 4. Discussion

The main purpose of the present study was to investigate neuronal networks for order processing in deaf and hearing individuals. We predicted general similarities across groups for both the digit and letter order tasks with some language modality-specific activation. Specifically, we hypothesized (1) general similar activation across groups at whole brain level, (2) significant activation for the digit order task in regions of interest in the parietal cortex across groups, (3) significantly greater activation for digit order compared to letter order in HIPS, reflecting magnitude specificity, and (4) significant group differences in the lAG for the digit order task, reflecting differential engagement of linguistic representations across groups. The results showed that there were general similarities across groups in relation to both task and regions but that none of our hypotheses was fully supported. Overall and in line with the previous studies [39], we found little neural activation in the deaf group due to substantial individual variability.

We predicted that the digit order task would activate bilateral parietal regions including the superior parietal cortex and the horizontal portion of the intraparietal sulcus in both groups. However, in the whole brain analysis, there was no evidence for either group of a general activation in bilateral parietal regions that has previously been attested for numerical ordering tasks. In fact, the digit order task versus visual control only elicited significant activation in the right cerebellum, when collapsed over groups. A meta-analysis conducted by Arsalidou and Taylor [40] of neuroimaging studies investigating numerical and arithmetic processing tasks showed that the cerebellum is generally activated for both number and calculation tasks. Arsalidou and Taylor [40] suggested that the role of the cerebellum was coordination of visual motor sequencing, which in the present study might be related to the visual inspection of the digit/letter string.

In the analysis performed on the five regions of interest based on our hypotheses, the only region to be significantly activated for the digit order task was the rHIPS for deaf signers. For hearing nonsigners, no significant activation was found. Hence, for the hearing group, we found no support for the notion of magnitude manipulation specificity of the horizontal portion of the intraparietal sulcus. Several studies [41] found that the mere presentation of single numbers compared to single letters generated activation in the intraparietal sulcus bilaterally, indicating automatic numerosity processing. The tasks in the present study (both experimental tasks and control task) were based on the same stimulus material to keep visual stimulation under control. Thus, any automatic number processing elicited simply by seeing numbers, even when they were not necessary for task solution, would have been removed by subtraction in the digit order versus visual control contrast. However, although there was no main effect of the group in the analysis of variances performed on the HIPS regions, deaf signers showed a significant activation of the rHIPS that was not found for the hearing group. This is in line with preliminary data from our lab, where we have found stronger activation for deaf signers compared to hearing nonsigners in the rHIPS in response to arithmetic tasks (using the same stimulus material as in the present study; [42]). Taken together, this indicates qualitatively different processes for deaf compared to hearing individuals relating to number processing. Notably, behavioural results did not differ significantly between groups despite activation of different brain regions. This suggests that deaf signers can make use of different brain regions compared to hearing nonsigners to reach the same goal.

In both the whole brain analysis and the ROI analyses, we found significant activation across groups for the letter order task within the visual processing system which not only included occipital regions but also extended into the parietal and frontal regions of the dorsal stream. This is in line with recent work showing more activation for letters than numbers in the left inferior and superior parietal gyri as well as a preferential role of the parietal cortex for letter identity and letter position encoding [43], indicating a prominent role for the dorsal processing stream in letter identification and position encoding [43, 44]. The greater engagement of the visual processing system for the letter order task compared to the digit order task probably reflects both the specificity of the visual system for letter processing and the greater demands of determining relative order of three items in a larger set. A recent study showed the sensitivity of earlier and later regions of the visual processing system to letter orientation and how this sensitivity develops from childhood to adulthood [45]. Further, while the set of Arabic digits used in the present study had ten items, the set of letters had 18 items (B, D, E, G, H, K, L, M, O, P, Q, T, U, V, X, Z, Å, and Ö) which in turn are part of the 29-item Swedish alphabet. Thus, the two experimental tasks used in the present study elicit two different kinds of ordering, one which requires numerical order processing within a small well-defined closed set and one that requires alphabetical order processing within a larger closed set, arbitrarily defined.

Interestingly, in the ROI analyses, the lSPL was significantly activated in hearing nonsigners, corroborating findings from previous studies suggesting the lSPL to have a central role in letter positioning [43, 44]. Deaf signers performed similarly to the hearing nonsigners on the letter order task but did not show any significant activation in the lSPL. Hence, for deaf signers, we found no evidence that lSPL engagement is necessary for letter ordering. However, we cannot exclude the possibility that the lack of lSPL engagement on this task underlies difficulties on higher level language tasks for this group. Lack of significant activation in this area has previously been found in a letter substitution task for individuals with dyslexia [44]. Although not investigated here, it is possible that the poorer reading ability generally found in deaf individuals [46] may stem from an inability to engage the lSPL in early letter processing.

Finally, we predicted that differential engagement of linguistic representations by deaf signers and hearing nonsigners during the digit order task would be reflected in differences in the activation of the lAG. Surprisingly, there was no significant activation in the lAG for either group and no significant differences in activation of this region related to either task or group. This region has been shown to be involved in verbal number processing, such as multiplication and simple subtraction [25, 33, 37]. The lAG has also been shown to play an important role in orthographic-to-phonological conversion [47], and so we had expected both our experimental tasks to engage this region. One explanation of our results is that both tasks are solved using nonlinguistic ordering manipulations rather than verbal processing.

## 5. Conclusion

The main finding of the present study is that there are similarities in the recruitment of neuronal networks during order processing in deaf signers and hearing nonsigners. The digit order task showed relatively little activation across groups possibly relating to the simplicity of the task. However, recruitment of the rHIPS in deaf signers only for this task suggests that compared to hearing nonsigners this group makes use of qualitatively different processes, such as magnitude manipulation for number order processing. Extensive activation of the dorsal stream relating to the letter order task indicates a prominent role for letter identification and position encoding. This finding prompts further investigation of the effects of deafness and sign language use on the neural networks underpinning core arithmetic processes.

## Figures and Tables

**Table 1:** Participant characteristics.

	Age			Sex	Education	Raven		
	*M*	SD	Range	Female/male	University^∗^	*M*	SD	Range
Deaf signers	28.1	3.44	21–32	11/5	5	52.3	5.13	44–60
Hearing nonsigners	28.5	4.78	22–37	12/5	5	54.7	4.04	45–59

^∗^Number of participants with university degree or equivalent education.

**Table 2:** Behavioural in-scanner data.

	Response time (ms)				Accuracy (% correct)			
	Deaf signers		Hearing nonsigners		Deaf signers		Hearing nonsigners	
	*M*	SD	*M*	SD	*M*	SD	*M*	SD
Digit order	1612	192	1603	314	96.0	6.87	98.6	1.86
Letter order	2345	327	2342	336	91.6	5.13	91.2	3.63

**Table 3:** Whole brain analysis. Activation foci for each contrast versus visual control for the two groups separately and combined. All peaks with significant activation are listed (*p*_fwe_ < 0.05).

Group	Task	Cluster level		Peak level		MNI coordinates			Brain region of the peak
		Size	*p*_fwe_	*T*	*p*_fwe_	*x*	*y*	*z*	
Deaf signers	Letter order	89	0.016	5.19	0.342	23	−72	44	r. superior occipital gyrus

Hearing nonsigners	Letter order	42	<0.001	9.10	0.004	16	−72	54	r. superior parietal lobule
				8.01	0.016	30	−72	39	r. middle occipital gyrus
		18	<0.001	8.90	0.005	30	9	59	r. middle frontal gyrus
		14	<0.001	8.89	0.005	−12	−75	49	l. precuneus
				7.30	0.043	−26	−68	54	l. superior parietal lobule
		6	0.001	8.37	0.010	−30	−79	34	l. middle occipital gyrus
		4	0.003	7.81	0.022	−54	−5	44	l. postcentral gyrus

Both groups combined	Digit order	5	0.006	6.01	0.010	23	−61	−36	r. cerebellum

Both groups combined	Letter order	137	<0.001	8.06	<0.001	23	−72	44	r. superior occipital gyrus
				7.87	<0.001	34	−68	34	r. middle occipital gyrus
		99	<0.001	7.65	<0.001	−26	−72	29	l. middle occipital gyrus
				7.20	0.001	−23	−68	49	l. superior parietal lobule
		31	<0.001	7.40	<0.001	27	6	54	r. superior frontal gyrus
		77	<0.001	7.14	0.001	−5	2	59	l. SMA
				6.93	0.001	−16	2	59	l. superior frontal gyrus
				6.69	0.002	−5	16	44	l. SMA
		18	<0.001	6.28	0.006	44	−40	44	r. inferior parietal sulcus
		2	0.015	5.58	0.030	−44	2	29	l. precentral gyrus
		1	0.023	5.39	0.045	−44	−47	44	l. inferior parietal sulcus

## Data Availability

The data used to support the findings of this study are available from the corresponding author upon request.
